# Near-Infrared Fluorescence Axillary Reverse Mapping (ARM) Procedure in Invasive Breast Cancer: Relationship between Fluorescence Signal in ARM Lymph Nodes and Clinical Outcomes

**DOI:** 10.3390/cancers14112614

**Published:** 2022-05-25

**Authors:** Muriel Abbaci, Angelica Conversano, Maryam Karimi, Marie-Christine Mathieu, Valérie Rouffiac, Frederic De Leeuw, Stefan Michiels, Corinne Laplace-Builhé, Chafika Mazouni

**Affiliations:** 1 UMS AMMICa, Plateforme Imagerie et Cytométrie, Gustave Roussy Cancer Campus, Université Paris-Saclay, 94805 Villejuif, France; valerie.rouffiac@gustaveroussy.fr; 2 Laboratoire d’Imagerie Biomédicale Multimodale Paris Saclay, Université Paris-Saclay, CEA, CNRS, Inserm, 91401 Orsay, France; 3 Department of Breast and Plastic Surgery, Gustave Roussy Cancer Campus, Université Paris-Saclay, 94805 Villejuif, France; angelica.conversano@gustaveroussy.fr (A.C.); chafika.menard@ch-eureseine.fr (C.M.); 4 Bureau de Biostatistique et d’Épidémiologie, Gustave Roussy Cancer Campus, Université Paris-Saclay, 94805 Villejuif, France; maryam.karimi@gustaveroussy.fr (M.K.); stefan.michiels@gustaveroussy.fr (S.M.); 5 Oncostat U1018, Inserm, Université Paris-Saclay, Équipe Labellisée Ligue Contre le Cancer, 94805 Villejuif, France; 6 Department of Pathology, Gustave Roussy Cancer Campus, Université Paris-Saclay, 94805 Villejuif, France; marie-christine.mathieu@gustaveroussy.fr

**Keywords:** indocyanine green, axillary reverse mapping procedure, fluorescence, breast cancer, lymph node, biophotonics

## Abstract

**Simple Summary:**

Near-infrared fluorescence axillary reverse mapping (ARM) is a promising procedure for identification and preservation of arm lymphatic drainage during axillary lymph node dissection (ALND). We included 109 patients to analyze the indocyanine green fluorescence signal in ARM lymph nodes after resection. The fluorescence signal from ARM lymph nodes were compared with clinical findings to determine the importance of this criterion on the potential management of patients with ALND. ARM lymph nodes were identified in 94.5% of cases. The mean normalized fluorescence signal intensity value was 0.47 with no significant signal difference between metastatic and non-metastatic ARM lymph nodes. Only the preoperative diagnosis of metastasis in the axillary nodes of patients was significantly associated with a higher ARM node fluorescence signal intensity. Although preliminary results did not show that fluorescence signal intensity is a reliable diagnostic tool, the NIR fluorescence ARM procedure may be useful for ARM lymph node identification.

**Abstract:**

The near-infrared (NIR) fluorescence axillary reverse mapping (ARM) procedure is a promising tool to identify and preserve arm lymphatic drainage during axillary lymph node dissection (ALND). The ARMONIC clinical trial was conducted to validate the technique on a large cohort of patients and to analyze the predictive clinical factors for ARM lymph node metastasis. For the first time, the fluorescence signal intensity from the ARM lymph nodes was measured and correlated with clinical findings. A total of 109 patients with invasive breast cancer and indications of mastectomy and ALND underwent the NIR fluorescence ARM procedure. Indocyanine green was administered by intradermal injection followed by intraoperative identification and resection of the ARM lymph nodes with NIR fluorescence camera guidance. The fluorescence signal intensity and signal distribution were then measured ex vivo and compared with clinical outcomes. ARM lymph nodes were successfully identified by fluorescence in 94.5% of cases. The mean normalized fluorescence signal intensity value was 0.47 with no significant signal difference between metastatic and non-metastatic ARM lymph nodes (*p* = 0.3728). At the microscopic level, the fluorescence signal distribution was focally intense in lymphoid tissue areas. Only the preoperative diagnosis of metastasis in the axillary nodes of patients was significantly associated with a higher ARM node fluorescence signal intensity (*p* = 0.0253), though it was not significantly associated with the pathological nodal (pN) status (*p* = 0.8081). Based on an optimal cut-off fluorescence value, the final sensitivity and specificity of the NIR fluorescence ARM procedure for ARM lymph node metastatic involvement were 64.7% and 47.3%, respectively. Although our preliminary results did not show that fluorescence signal intensity is a reliable diagnostic tool, the NIR fluorescence ARM procedure may be useful for ARM lymph node identification. Clinical trial registration: NCT02994225.

## 1. Introduction

During operations, the decisions of surgeons are mainly guided by eyes and tissue palpation. Near-infrared fluorescence (NIR) imaging is the most promising method of real-time surgical imaging support [1,2]. Optical imaging in the NIR light spectrum with λ-excitation >700 nm has many advantages: increased tissue penetration of light, no endogenous fluorescence, less scattering and augmented signal-to-background ratio [3]. The potential of NIR fluorescence-guided surgery for multiple applications, such as tumor tissue resection, lymph node identification and functionality assessment (lymph flow visualization), has been described over the past 15 years [4].

Indocyanine green (ICG) is a heptamethine cyanine fluorophore that was developed in 1955 by Kodak photography; it received FDA approval in 1959 [5]. ICG has been the only NIR fluorophore clinically approved for ophthalmic angiography to determine cardiac output, hepatic function and liver blood flow for several decades; however, in France, its oncological use is still unlicensed. ICG dye has the advantages of being nonionizing and safe as well as allowing real-time optical guidance during surgery [6]. The maximum spectral absorption of ICG is 800 nm (between 780 nm to 805 nm) and its emission peak is at 820 nm, allowing for fluorescence imaging without autofluorescent background noise. Allergic reactions occur in fewer than 1 in 10,000 cases after systemic injection [7]. Although ICG is a non-selective molecule for breast cancer, it has been demonstrated to spread in more highly vascularized tissues, as metastasis can. Extravascular accumulation of ICG is responsible for an observed high fluorescence signal in tumoral tissue compared to the surrounding normal tissue. Few studies have described lymph node imaging after ICG intravenous injection [8,9,10] to identify metastatic lymph nodes. Bennett et al. previously suggested that involved lymph nodes and uninvolved lymph nodes could be discriminated based on the intensity and spatial localization of the fluorescence signal after intravenous injection of ICG-conjugated ultra-pH-sensitive nanoparticles into a mouse model with the characterization of microscopically patterned nanomaterial accumulation in lymph nodes [11]. Although the fluorescent dye is commonly injected systemically, subcutaneous injection has also been successfully reported for fluorescence imaging of lymphatic ducts and lymph nodes [12,13]. Kitai et al. first used ICG for fluorescence visualization of lymphatic channels and sentinel lymph nodes (SLNs) in breast cancer after subareolar injection [14].

After axillary lymph node dissection (ALND), the incidence of lymphedema is between 7 and 77% [15,16,17,18,19]. In 2007, axillary reverse mapping (ARM) was proposed by Thomson et al. [20] and Nos et al. [21] to identify and preserve arm lymphatic drainage during ALND. According to the theory that the arm and the breast each have specific lymphatic drainage pathways, ARM lymph nodes are identified by subcutaneous or intradermal injection of a tracer into the arm prior to surgery, followed by a brief massage and elevation of the arm [19]. Then, the tracer diffuses through the lymphatic ducts to the axillary lymph nodes draining the arm [22]. First described by Noguchi et al. [23], the use of NIR fluorescence imaging combined with ICG injection has been reported in breast cancer for ARM node identification and lymphatic visualization in many clinical studies, but whether the fluorescence signal intensity correlates with clinical outcomes has not yet been analyzed [19,24].

We conducted a clinical trial named ARMONIC (Axillary Reverse Mapping Using Near-Infrared Imaging in Invasive Breast Cancer study) to determine the rate of identification of the reverse axillary node using ICG and NIR fluorescence imaging in invasive breast cancers that underwent total mastectomy and ALND as the primary objective. The secondary objective was to identify predictive factors for metastatic ARM nodes and to analyze oncological concerns.

In this paper, we analyze the fluorescence signal in ARM lymph nodes after resection for the first time. The fluorescence signal from ARM lymph nodes was compared with clinical findings to determine the importance of this criterion on the potential management of patients with ALND. Fluorescence signal distribution in the ARM lymph node was also explored at the microscopic level.

## 2. Material and Methods

### 2.1. Patient Characteristics

One hundred and nineteen women diagnosed with primary invasive breast carcinoma who had an indication for mastectomy and ALND according to French guidelines at the time of study approval were included. Patients were prospectively enrolled from March 2017 to June 2019, at Gustave Roussy Cancer Campus, Villejuif, France. Patients with distant metastases, inflammatory breast cancer, previous axillary surgery or hypersensitivity to ICG were excluded from the study. The ARMONIC protocol was conducted in accordance with the Declaration of Helsinki. The Gustave Roussy institutional review board approved the prospective study and informed consent was obtained from all patients. Of the 119 initially enrolled in the study, 8 withdrew consent, 1 was not included because of disease progression, and the surgical indication for 1 patient changed. The final cohort comprised 109 patients.

Clinical trial registration: NCT02994225, first registration: 10 March 2017.

### 2.2. Surgical Procedure

The patients received standard surgical treatment for their primary breast cancer with mastectomy and ALND according to ESMO Clinical Practice Guidelines on Breast Cancer and French guidelines at the time of the study (2017–2019). ALND was performed through the mastectomy incision or a separate incision, in the case of a skin-sparing mastectomy with immediate breast reconstruction. ALND encompassed lymph nodes from levels I and II. According to French guidelines and NCCN guidelines version 5.2021, a level III dissection should be performed only in cases with gross disease in level II and/or III.

### 2.3. In Vivo ARM Lymph Node Identification

NIR fluorescence ARM procedure was achieved as such:

- Before the mastectomy incision, 1 mL of 2.5 mg/mL of ICG (Infracyanine, Serb Paris, Paris, France) was injected intradermally in the ipsilateral upper extremity in the second interdigital space (0.5 mL) and then on the inner face of the elbow (0.5 mL). The protocol was based on two injection sites to improve the ICG’s circulation through the lymphatic vessels. The choice to perform a double site injection was based on published protocols that intended to localize the ARM node as previously described [24]. We did not massage directly after the first injection so as to not overwhelm the fluorescent dye diffusion. The volume of ICG injected to identify the ARM node was the same for all patients, so all included cases received 2.5 mg of ICG.
- Ten minutes after injection, the presence of ICG in the upper limb was detected with a Spectrum^®^ hand-held NIR fluorescence camera (Quest medical imaging, Middenmeer, the Netherlands) [25]. If the fluorescence signal was quickly observed on the forearm/arm, the surgeon did not add any massage. However, when the signal remained only at the injection sites, the surgeon massaged the corresponding area for a few seconds. The course of the intradermal lymphatic ducts could be delineated from the ICG fluorescence signal on the skin from the hand to the forearm and finally to the axilla.
- The skin incision for mastectomy was initiated 15 min after the initial injection of ICG dye.
- After mastectomy and during ALND, fluorescent lymph nodes were identified by the surgeon and removed separately. Their localization in the axillary basin and the time of removal was noted precisely to determine the most frequent ARM lymph node location and time interval for removal. The axillary basin was anatomically divided into four zones (A, B, C and D) by the lateral thoracic vein (LTV) vertically and the second intercostobrachial nerve (ICBN) horizontally as described in [26].
- The removed ARM lymph nodes were isolated from the rest of the axillary lymph nodes for further pathology examination.

### 2.4. Fluorescence Data Analysis Procedure

The fluorescence signal of freshly isolated ARM lymph nodes was measured just after resection with a Spectrum^®^ hand-held NIR fluorescence camera. The head of the camera was at a fixed focal distance of 15 cm to image the ARM lymph nodes ex vivo, and the conditions of data acquisition were consistent throughout: laser power 100%, time exposure 30 ms and gain 15.4 dB for an optimal signal detection without fluorescence signal saturation. As a positive standard, a 2.5 mL syringe filled with ICG diluted at 5 µg/mL was used to normalize the fluorescence signal value (ARM lymph node fluorescence signal value/standard fluorescence signal value). Diluted ICG solution was chosen as the reference for quantitative analysis in the ARMONIC protocol to standardize ex vivo data acquisition in the operating room (same working distance, incident angle and settings). A new standard was processed for each patient inclusion. The calculated fluorescence signal ratio called “normalized fluorescence signal intensity value” was the main criterion.

### 2.5. Histological Analysis

All lymph nodes found during the ALND were isolated, sliced into two equal parts and embedded in paraffin, cut on one level of 3 µm thickness and stained with hematoxylin-eosin-saffron (HES).

ARM lymph nodes were embedded in paraffin, serially sectioned in slices of 2–3 µm that were cut on four levels at 200 µm intervals and stained with HES and by immunohistochemistry for cytokeratin (AE1/AE3).

Nodal metastases were classified according to UICC guidelines. The total number of isolated lymph nodes, the number of metastatic lymph nodes and the size of the metastases (micro-metastases <2 mm or macro-metastases >2 mm) were reported for the ALND and ARM lymph nodes. ARM nodes containing macro- or micro-metastases were considered positive, whereas those containing isolated tumor cells were considered negative.

Histological sections from ARM nodes were scanned at 20× magnification with a NanoZoomer S210 Scanner (Hamamatsu Photonics, Massy, France) for data analysis and archiving. NDPI format was converted into Tiff format with ImageJ plugin “NDPItools” for subsequent analysis.

### 2.6. Paraffin Block Imaging

After sectioning of the paraffin-embedded ARM lymph nodes for HES staining, the fluorescence imaging of ARM lymph nodes and control lymph nodes (from patients with ALND but without ICG injection) in paraffin blocks was achieved as follows: lymph nodes were scanned for spatial fluorescence distribution on an IVIS Spectrum CT scanner (PerkinElmer^®^, Villebon sur Yvette, France). The 2D fluorescent images were acquired in epi-illumination (top-down) using the adequate filters for ICG detection (excitation 745 nm ±15 nm and emission 820 nm ±10 nm). Acquisition settings were automatically computed by the system to optimize the signal detection according to the fluorescence level for each lymph node. Fluorescence intensity was expressed as radiant efficiency, which is a normalized expression of the fluorescence according to acquisition settings. Thus, fluorescence distributions can be compared between lymph nodes as intensities are normalized and coded with a unique color scale for all lymph nodes represented on the same image.

The fluorescence distribution and white-light image from the ARM lymph nodes in the paraffin blocks were then overlapped with corresponding digital HES sections (last level section) using Adobe Photoshop software CS6 13.0. The data were presented to a pathologist to determine the fluorescence signal distribution in the ARM lymph node sections at the microscopic level.

### 2.7. Statistical Analysis

The Spearman’s rank correlation coefficient test was used to measure the correlation between the time from ICG injection to ARM node identification and the normalized fluorescence value. The Wilcoxon rank-sum test was used to investigate whether the technical procedure’s success was linked to body mass index (BMI) and patient age, as well as whether the observed fluorescence values were significantly different according to variable levels among the patients who received ALND. A 5% significance level was used, and all *p* values were two-sided. The Pearson correlation was used to test whether there is a statistically significant linear relationship between mean fluorescent values and BMI among the patients for whom the technique did not fail. To determine the diagnostic accuracy of NIR fluorescence axillary reverse mapping in involvement detection, the mean fluorescence signal intensities of the ARM lymph nodes were categorized using an optimal threshold value of 0.40 after testing sequential values from 0.1 to 0.6 [27]. With this categorization, we then calculated the sensitivity (defined as the proportion of patients with positive fluorescent nodes that had a mean fluorescence signal intensity ≥0.4 = TP/P), specificity (defined as the proportion of patients with negative fluorescent nodes that had a mean fluorescence signal intensity <0.4 = TN/N), positive predictive value (defined as the proportion of patients with truly positive fluorescent nodes among those that had a mean fluorescence signal intensity ≥0.4 = TP/(TP + FP)), negative predictive value (defined as the proportion of patients with truly negative fluorescent nodes among those that had a mean fluorescent signal intensity <0.4 = TN/(TN + FN)) and accuracy (defined as the proportion of true positive and true negative in all cases = (TP + TN)/N) with their confidence intervals using the Clopper–Pearson (also known as exact interval) method. The cut-off value was determined with the method focused on sensitivity and specificity values described by Habibzadeh et al. [28].

All analyses were performed using the SAS analysis software, version 9.4 (SAS Institute, Cary, NC, USA).

## 3. Results

### 3.1. Fluorescent ARM Node Procedure

**Figure 1:**
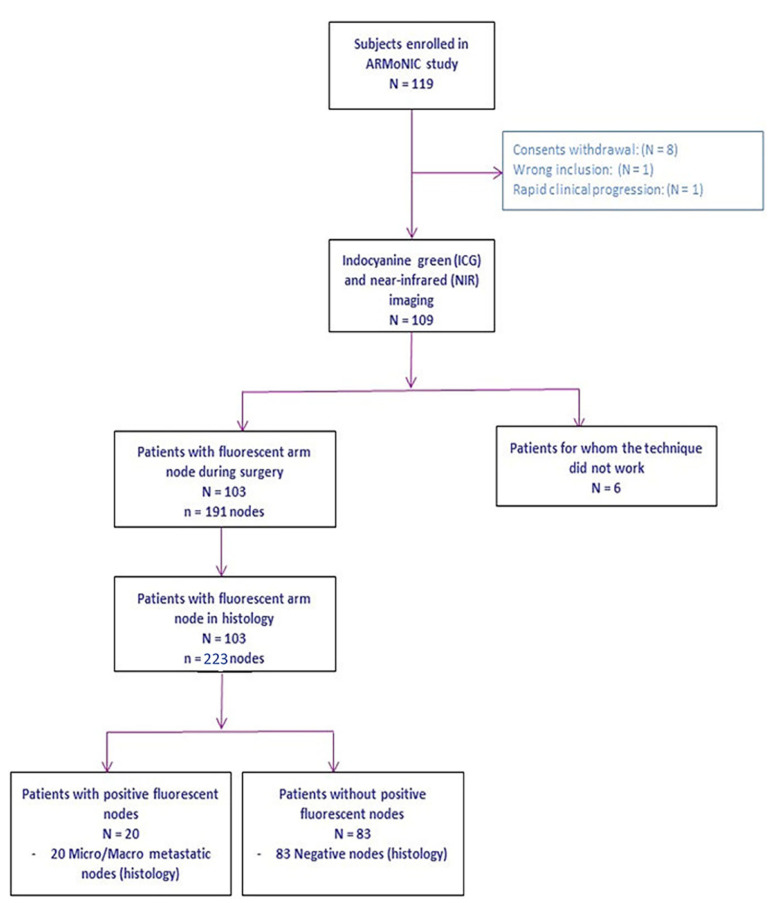
Flow chart of ARMONIC protocol.

ICG was administered to 109 patients and NIR fluorescence imaging was then achieved during ALND to identify ARM lymph nodes. Characteristics of the study population are presented in Table 1. The CONSORT diagram for the trial is shown in Figure 1.

**Figure 2:**
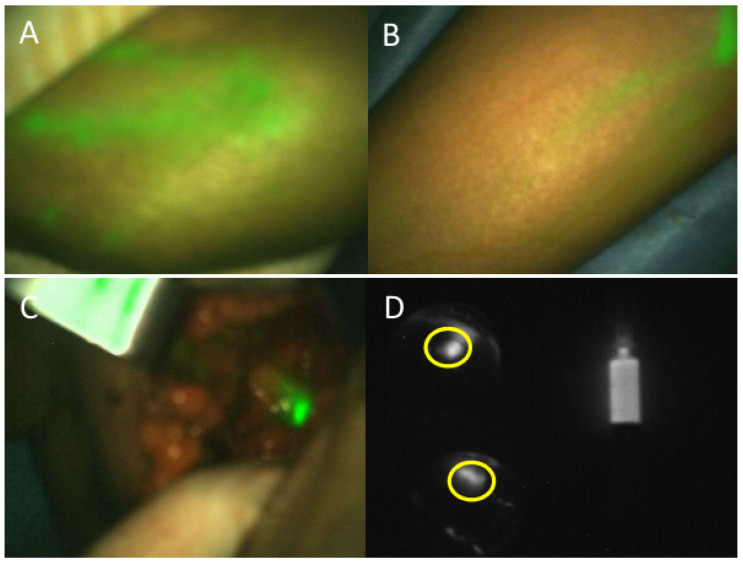
Intraoperative fluorescence axillary reverse mapping procedure during mastectomy completed by axillary lymph node dissection. (**A**) Ten minutes after ICG intradermic injection, the fluorescent signal (in green) can be observed in lymphatics ducts over the hand. (**B**) Fluorescence signal can be followed along the arm. (**C**) In vivo fluorescence signal from ARM lymph node detected inside the incision. (**D**) Ex vivo fluorescence imaging to measure fluorescence signal from two ARM lymph nodes (in yellow circle) and signal in the fluorescence standard.

Fluorescent lymphatic ducts were visible in the forearm in more than 83.3% patients and in the upper arm in more than 66.7% patients before skin incision (Table 2 and Figure 2A,B). Fluorescent ARM node detection and resection was quick, simple and effective for 103 (94.5%) patients (Figure 2C). ARM lymph nodes were not detected in six patients, and this was not correlated with BMI (*p* = 0.7304) or patient age (*p* = 0.1045). The mean duration between the ICG injection and ARM lymph node identification was 59 min (SD 24–100) (Table 2). Final histology confirmed the resection of 223 ARM lymph nodes with a median of 2 per patient. Of 103 patients with a successful ARM procedure, 55 had metastatic axillary lymph nodes in the final histology, and 20 had metastatic ARM lymph nodes. Eighteen patients had both metastasis-positive ARM nodes and ALND. Two patients had only metastatic ARM lymph nodes but not in the rest of the axillary lymph nodes. Of all 223 ARM lymph nodes, 195 had no tumor cells, 3 had isolated tumor cells, 3 had micro-metastases, 15 had macro-metastases and 7 had macro-metastases with extracapsular invasion.

No serious adverse events were reported for all the included patients.

### 3.2. Fluorescent ARM Node Signal Analysis

In vivo, we observed the fluorescence signal from ARM lymph nodes, from lymphatics to the ARM nodes and from ICG leakage in axilla after ARM lymph node resection (fluorescence puddle).

Shortly after ARM lymph node resection, we measured ex vivo the fluorescence signal from the ARM lymph nodes of 91 patients (Figure 2D). At the time of resection, surgeons macroscopically identified 191 lymph nodes, which was refined to 223 lymph nodes during the final histology. In some cases, the surgeons misinterpreted a fresh, small cluster of lymph nodes as one big lymph node. The differentiation could be completed on final HES sections leading to a higher final number of ARM lymph nodes. A total of 12 non-consecutive patients could not be included in this part of the study as the measurement conditions were inconsistent with the remaining 91 patients (140 lymph nodes), mainly due to variations in acquisition settings, which were identified at the time of data analysis.

For each patient, we considered the mean observed fluorescence signal values from the ex vivo ARM lymph nodes and compared these values with clinical findings (Table 3). The mean fluorescence signal value for all of the imaged ARM lymph nodes was 0.47 (SD 0.27). There was no linear relationship between the fluorescence intensities of ARM lymph nodes and BMI (*p* = 0.1392). There also was no correlation between time from the ICG injection to ARM node identification and the normalized fluorescence value (ρ = −0.034). The mean fluorescence signal value of the ARM lymph nodes was significantly higher when patients had metastasis in the axillary nodes at the time of preoperative diagnosis: 0.52 versus 0.43 (*p* = 0.0253). However, the fluorescence signal intensities of the ARM lymph nodes were not significantly affected by whether the patients had received chemotherapy.

Although the ex vivo fluorescence signal in the ARM lymph nodes was 1.4 times higher when the axillary lymph nodes were histologically positive for metastases (pN+), the difference was not significant. We also noticed no significant fluorescence signal difference between metastatic and non-metastatic ARM lymph nodes.

We noted the anatomical localization of the ARM lymph nodes at the time of resection [26]: 63.4% of ARM nodes were in the D zone, 25.6% in the B zone, 7.3% in C zone, 2.1% in A zone and 3% could not be determined. Table 3 reports the mean fluorescence intensity of the ARM lymph nodes per zone.

We tested sequential cut-off values to classify the normalized fluorescence values according to metastatic status of the ARM lymph node. With an optimal cut-off at 0.4, we calculated a sensitivity of 64.7%, a specificity of 47.3% and an accuracy of 50.5%, respectively (Table 4).

All patients had a postoperative clinical examination one month after surgery. At the time of the examination, 10.9% of patients presented lymphocele. The ARM lymph node fluorescent signal intensities at the time of ALND were not significantly different in patients with and without lymphocele one month after surgery.

### 3.3. ICG Fluorescence Distribution in ARM Lymph Node at the Microscopic Level

**Figure 3:**
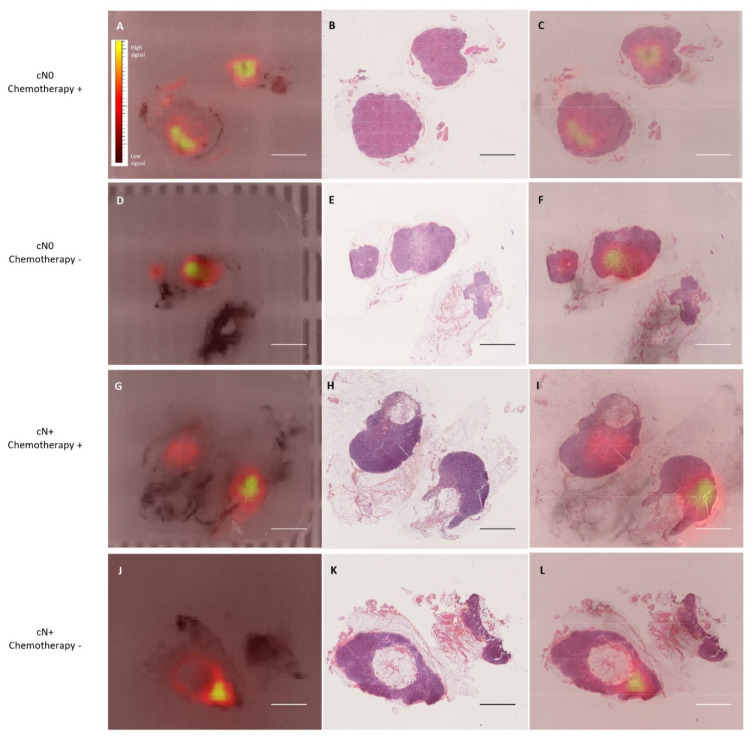
Fluorescence signal distribution in fixed and cut ARM lymph nodes according to preoperative diagnosis of axillary nodal status and preoperative chemotherapy. (**A**,**D**,**G**,**J**) are macroscopic fluorescence imaging from ARM lymph nodes in paraffin block. (**B**,**E**,**H**,**K**) are corresponding HES section. (**C**,**F**,**I**,**L**) are overlay of fluorescence signal with HES sections (Scale bar 5 mm and color scale for fluorescence signal intensity)).

We analyzed the fluorescence signal distribution in ARM lymph nodes after tissue fixation and paraffin-block cutting, as previous articles reported a persistence of the fluorescence signal from ICG in fixed tissues [29]. The fluorescence signal was visible in all blocks imaged by IVIS Spectrum scanner (Figure 3A,D,G,J). A negative control group confirmed that the fluorescence signal originated only from the ICG dye. When the fluorescence images overlapped with the corresponding HES-stained section (Figure 3B,E,H,K), we noticed a focal fluorescence signal in all of the analyzed ARM lymph nodes that was independent of chemotherapy treatment, preoperative diagnosis of axillary nodal status, pN status or ARM lymph node metastasis. The fluorescence signal was always localized in lymphoid tissue under the capsule (Figure 3C,F,I,L).

## 4. Discussion

Both surgeons (AC and CM) were familiar with the fluorescent SLN procedure using a hand-held NIR fluorescence camera in breast cancer surgery [30]. The transposition of the same skills to perform ARM was a natural progression. The training to inject ICG efficiently was short (one patient). The surgeons were experienced after only few procedures, allowing for identification and resection of the fluorescent ARM lymph node from lymphatic leakage and vessels around the axillary and thoracic veins with a maximum surgery duration increase of 10 min. The NIR fluorescence procedure was successful with a detection rate for ARM nodes of 94.5%, a higher rate than in the existing literature (80–88%) [19]. In previous studies, we noted a great variability in the injection volume and final dose of the ICG injected from 0.1 mL corresponding to 0.25 mg of ICG to 1 mL corresponding to 5 mg of ICG injected but limited to one injection site: interdigital, wrist, upper inner arm or proximal arm at the intermuscular groove [19]. Here, we injected in the second interdigital space (0.5 mL) and then on the inner face of the elbow (0.5 mL), corresponding to a total of 2.5 mg of ICG that could be optimal for the NIR fluorescence ARM procedure. The injection technique may influence the results due to inherent variations in lymphatic anatomy. This could be further explored in a larger series to identify the most appropriate site for injection. The Spectrum hand-held NIR fluorescence camera was also of interest when performing axillary cavity images as we could overlay in live fluorescence signal and white light images on the screen during surgery. Most of the previous studies were reported with the PDE system’s former version, which was the first generation of NIR fluorescence cameras leading to less sensitivity for detection of the fluorescence signal. The standardization of data acquisition during fluorescence quantification was mandatory. As described by Heeman et al. in a guideline for surgeons interested in fluorescence guided surgery, the choice of the imager affects the camera’s detection sensitivity to the required dye, depth sensitivity, field illumination homogeneity, spatial and temporal resolutions and dynamic range [31]. The camera settings had to be defined prior the 103 patients’ inclusion to optimize the signal-to-noise ratio during data acquisition. We can finally report that the ARM procedure was described in some studies during the SLN procedure. The difference in rate detection between ALND and SLN for ARM node identification may be explained by a reported localization of the fluorescent ARM nodes mainly between the axillary vein and the second intercostobrachial nerve and close to the anterior edge of the latissimus dorsi muscle, regardless of the contrast agent injected, as described by Ikeda et al. [32]. Not only was the fluorescence technique useful for detecting the ARM lymph nodes, but also for detecting the lymphatic ducts (66.7–99% of upper arm and forearm imaging), as previously described by Noguchi et al. [33]. In contrast to Tausch et al. [34], we did not observe a failure to detect ARM lymph nodes when there was a high tumor burden in the axilla. For the six unsuccessful fluorescence ARM procedures, two patients had fluorescence signals in lymphatic vessels in the axilla at the time of ALND, and the fluorescent tissue was resected; however, the final histology detected no lymphoid tissue. Three patients had fluorescent lymphatic ducts in the arm but not in the axilla, and one patient had no fluorescent signal outside the ICG injection sites. In previous ARM procedures, ICG was injected from 2 h before surgery to anesthesia time, followed by the surgery 5 min after. Given the lack of standardization of the protocol and the dose of ICG to be injected, an objective comparison of the clinical results may not be relevant, based on the criterion of timing of the surgical procedure [19]. Noguchi et al. reported a cross over rate of SLN-ARM in 28% of cases with patients with cN0 who underwent SLN biopsy and ARM procedure. An overlay of SLN and positive ARM nodes would have been observed in the ARMONIC population if the SLN procedure had been achieved before ALND.

For the first time in a clinical trial with NIR fluorescence ARM procedure, the fluorescence signal intensity was measured in ARM lymph nodes of 91 patients and normalized to allow for intra- and interpatient comparison. We found that the involvement of axillary lymph nodes at preoperative diagnosis was correlated with the fluorescence intensity of the ARM node at the time of resection, which was independent of preoperative chemotherapy. Surprisingly, although the fluorescence intensity was higher in the metastatic ARM lymph nodes, compared with the non-metastatic ARM lymph nodes, these data were not statistically significant. In addition, the fluorescence level of the ARM lymph nodes at the time of determination of final axillary lymph node involvement was not linked to the final metastatic axillary lymph node diagnosis. The process for preferential uptake of ICG in tumor tissues is not entirely understood. The most likely proposition includes the enhanced permeability and retention effect observed in tumoral tissue after neoangiogenesis [29]. Recently, Bourgeois et al. analyzed the fluorescence signal in breast surgical specimens, as well as the SLN and ALND after ICG intravenous injection [8]. In the case of fluorescence signal in the lymph nodes after ALND, they proposed two hypotheses to explain ICG staining: fluorescent dye accumulation through the metastatic tumor tissues of the involved lymph nodes and dye loading in non-metastatic lymph nodes when in an inflammatory state [8]. Xia et al. and Digonnet et al. also measured the fluorescence signal intensity in SLN or cervical lymphadenectomy after intravenous or peritumoral ICG injection in head and neck cancer studies [9,35]. They reported a higher fluorescence signal in metastatic lymph nodes. In the case of identification of metastatic lymph nodes for ovarian cancer, Pop et al. also proposed, after intravenous ICG injection, the application of a cut-off to the ratio of the fluorescence signal to discriminate the involved and not-involved lymph nodes [27]. A robust cut-off value could then be suggested to guide surgeons in the resection of decisive lymph nodes. In their study, they calculated that a fluorescence ratio of 1.3 or more was associated with a 2.64-fold increased risk of having a metastasis-positive retroperitoneal node. We also proposed an optimal cut-off value (0.4) for ARM lymph node diagnosis. Our results on sensitivity and specificity show that the fluorescence signal intensity in an ARM lymph node after ICG injection cannot be used as a criterion to consider during conservation of the lymph node draining the arm during ALND. Multi-wavelength fluorescence imaging with a dual-band imager could allow for preservation of the ARM node after fluorescent dye injection (functionality lymphatic pathway assessment) and resection of metastatic ARM lymph nodes during ALND with the concomitant administration of a fluorescent specific molecule [36]. The need for “live molecular navigation”, which requires molecules that both localize specific target tissues, such as breast cancer, and are able to spread and perfuse in the living patient [4,37], has prompted researchers to experiment with new tumor-specific targeting agents using fluorescence imaging. Most of them are under phase I or II and investigations are made particularly for lumpectomy [38]: it could be interesting to try one of these for the ARM procedure to get a high sensitivity and specificity.

In this prospective clinical study, we resected ARM lymph nodes usually one hour after the first ICG injection. Although we could expect a homogeneous distribution of ICG dye in lymphoid tissue at the microscopic level, the focal signal might represent the entry point of the dye at the time of resection. In physiology, the lymph travels to the lymph nodes, entering through afferent lymphatic collecting vessels. Then, the lymph flows around the outer portion of the lymphatic lobules through the subcapsular sinus [39]. The focal fluorescence signal distribution in ARM lymph nodes may represent the progressive input of the fluorescent dye through lymphatic ducts into the lymph node under the capsule. Here, ICG could be compared to lymph fluid circulation from the hand to the axilla into the arm lymph nodes. Without dissection of the identified fluorescent lymph node, the fluorescent dye will diffuse by the lymphatics along the lymph node network, which will also become fluorescent. The overlay of the fluorescent signal distribution with the HES section gave us a screenshot of the entry of the dye in the ARM lymph node at this time point.

## 5. Conclusions

Although NIR fluorescence ARM procedure is relevant for arm lymph node identification, the fluorescence signal intensity may not be used as a diagnosis tool to consider the conservation of the arm lymph node. In this study, patients were not followed long term to correlate the fluorescence signal intensity in the ARM node with the diagnosis of lymphoedema. However, this clinical evolution can be compared to the fluorescence intensity in the ARM node at the time of dissection to determine whether fluorescence signal intensity in ARM nodes could be a predictive factor of subsequent lymphoedema.

## Figures and Tables

**Table 1:** Characteristics of the study population.

Characteristics	*n* = 109	
Age, median (range)	53 (25–93)	
BMI, median (range)	25 (16–39)	
Clinical tumor node staging		
T1	15 (13.8%)	
T2	44 (40.4%)	
T3	48 (44%)	
T4	2 (1.8%)	
N0	53 (48.6%)	
N1	52 (47.7%)	
N2	3 (2.8%)	
N3	1 (0.9%)	
Pathological tumor node staging	pTpN (*n* = 52)	ypTypN (*n* = 57)
T0	0 (0.0%)	19 (17.43%)
Tis	0 (0.0%)	5 (4.59%)
T1	12 (11.01%)	11 (10.09%)
T2	31 (28.44%)	13 (11.93%)
T3	7 (6.42%)	7 (6.42%)
T4	2 (1.83%)	1 (0.92%)
Tx	0 (0.0%)	1 (0.92%)
N0	19 (17.43%)	30 (27.52%)
N1	21 (19.27%)	15 (13.76%)
N2	9 (8.26%)	8 (7.34%)
N3	3 (2.75%)	4 (3.67%)
Final histology (*n* = 90)		
Lobular	21 (23.3%)	
Ductal	62 (68.9%)	
Mixed	1 (1.1%)	
Other carcinoma	6 (6.7%)	
Presence of ductal carcinoma in situ	55 (61.1%)	

**Table 2:** NIR fluorescence ARM procedure results for *n* = 109 patients.

NIR Fluorescence ARM Procedure Results	Identification of Fluorescent ARM Lymph Node	
	Yes (*n* = 103)	No (*n*= 6)
% patients with fluorescent lymphatic vessels in forearm	99%	83.3%
% patients with fluorescent lymphatic vessels in upper arm	72.8%	66.7%
Time from ICG injection to ARM node identification in minutes, median (range)	59 (24–100)	-
Total number of identified ARM lymph nodes in final histology	223	-
Median per patient (Q1; Q3)	2 (1; 3)	-
Min; Max	1; 6	-
Number of patients with ex vivo fluorescence lymph node signal analysis	91	-
Number of patients with metastatic axillary lymph node in final histology	55	5
Number of patients with metastatic ARM lymph node in final histology	20	-
ARM lymph node final histology		
Isolated tumor cells	3	-
Micro-metastases	3	-
Macro-metastases	22	-
Capsular breakage	7	-
No tumor cells	195	-

ARM: axillary reverse mapping.

**Table 3:** Mean fluorescence signal value per clinical findings (*n*= 91 patients).

Clinical Findings	Mean Normalized Fluorescence Value (SD)	*p* Value
ARM lymph nodes	0.47 (0.27)	-
Preoperative diagnosis		0.0253
cN+ (*n* = 47)	0.52 (0.25)	
cN0 (*n* = 44)	0.43 (0.29)	
Preoperative chemotherapy		0.8457
yes (*n* = 47)	0.47 (0.26)	
no (*n* = 44)	0.48 (0.29)	
Per zone ARM lymph node identification		0.6489
A (*n* = 5)	0.48 (0.49)	
B (*n* = 32)	0.54 (0.37)	
C (*n* = 10)	0.47 (0.37)	
D (*n* = 84)	0.42 (0.25)	
Undefined (*n* = 9)	0.44 (0.29)	
ARM lymph node involvement		0.3728
yes (*n* = 17)	0.54 (0.32)	
No (*n* = 74)	0.46 (0.26)	
Axillary lymph node involvement		0.8081
pN+ (*n* = 49)	0.48 (0.27)	
pN0 (*n* = 42)	0.47 (0.28)	
Post-operative lymphocele		0.8045
yes (*n* = 10)	0.44 (0.21)	
No (*n* = 81)	0.48 (0.28)	

ARM: axillary reverse mapping.

**Table 4:** Diagnostic accuracy of NIR fluorescence in ARM lymph nodes’ involvement with cut-off fluorescence value of 0.4 (*n* = 91 patients).

Cut-off Value	Sensitivity (95% CI)	Specificity (95% CI)	Accuracy (95% CI)	NPV (95% CI)	PPV (95% CI)
Metastatic if mean fluorescence value ≥0.4 Negative if mean fluorescence value <0.4	64.7% (38.3–85.8)	47.3% (35.6–59.3)	50.5% (39.9–61.2)	85.4% (70.8–94.4)	22.0% (11.5–36.0)

NIR: near-infrared; ARM: axillary reverse mapping; NPV: negative predictive value; PPV: positive predictive value.

## Data Availability

Data from this clinical trial are available from the authors and can be requested by filling out the data request form for Gustave Roussy clinical trials at https://redcap.gustaveroussy.fr/redcap/surveys/?s=DYDTLPE4AM. The trial steering committee and the sponsor will review the requests on a case-by-case basis. In case of approval, a specific agreement between the sponsor and the researcher may be required for data transfer.
